# Aqueous extract of *Hibiscus sabdariffa* inhibits pedestal induction by enteropathogenic *E*. *coli* and promotes bacterial filamentation *in vitro*

**DOI:** 10.1371/journal.pone.0213580

**Published:** 2019-03-08

**Authors:** Reda Mohamed-Salem, Carmina Rodríguez Fernández, Elvira Nieto-Pelegrín, Beatriz Conde-Valentín, Angel Rumbero, Narcisa Martinez-Quiles

**Affiliations:** 1 Department of Microbiology and Parasitology, Pharmacy School, Complutense University, Madrid, Spain; 2 Pharmaceutics and Food Technology Department, Complutense University, Madrid, Spain; 3 Organic Chemistry Department, Autonóma University, Madrid, Spain; 4 Department of Immunology, Ophthalmology and ENT, School of Medicine, Complutense University and Gregorio Marañón Health Research Institute (IiSGM), Madrid, Spain; VIT University, INDIA

## Abstract

Diarrheic diseases account for the annual death of approximately 1.9 million children under the age of 5 years, and it is a major cause of work absenteeism in developed countries. As diarrheagenic bacteria, enteropathogenic *Escherichia coli* (EPEC) attach to cells in the small intestine, causing local disappearance of microvilli and inducing the formation of actin-rich pedestals that disrupt the intestinal barrier and help EPEC adhere to and infect intestinal cells. Antibiotics and other bioactive compounds can often be found by analyzing traditional medicines. Here a crude aqueous extract of *Hibiscus sabdariffa*, which typically grows in subtropical and tropical areas and is a popular medicinal tisane in many countries, was analyzed for antibacterial activity against EPEC. In standard microdilution assays, the extract showed a minimum inhibitory concentration of 6.5 mg/ml against EPEC growth. Time-kill kinetics assays demonstrated significant 24 h bactericidal activity at 25 mg/ml. The extract is able to impede pedestal induction. Not only did the extract inhibit preformed pedestals but it prevented pedestal induction as well. Remarkably, it also promoted the formation of EPEC filaments, as observed with other antibiotics. Our results *in vitro* support the potential of *Hibiscus sabdariffa* as an antimicrobial agent against EPEC.

## Introduction

Pathogenic strains of *Escherichia coli*, which are broadly categorized as either diarrheagenic or extraintestinal [1–3], have acquired virulence factors that provide them with the ability to cause a broad range of diseases in humans. Among diarrheagenic *E*. *coli*, enteropathogenic *E*. *coli* (EPEC) is an important cause of food- and water-borne diarrhea illness worldwide, especially in developing countries [4]. EPEC infection results in diarrhea, vomiting, fever and even death, contributing to 10–40% of deaths due to diarrhea among children under the age of five years around the world [5, 6]. Thus EPEC is one of the predominant diarrheagenic *E*. *coli* in clinical isolates from children [7, 8]. It is a non-invasive bacterium that adheres to small bowel enterocytes and destroys the normal microvillus architecture, inducing the formation of characteristic attaching-and-effacing (A/E) lesions and cytoskeletal changes. These changes include accumulation of polymerized actin structures called pedestals beneath the adherent bacteria [9].

After initial adherence to the cell surface, EPEC uses a type III secretion system to deliver effectors into host cells. One such effector is the translocated intimin receptor (Tir), which drives the major pathway responsible for regulating actin polymerization. Tir inserts in the plasma membrane and binds the bacterial adhesin intimin [10]. This binding is accompanied by the clustering and phosphorylation of Tir, which recruits many host cell proteins including Nck, which in turn recruits N-WASP [11] and other actin-nucleating proteins such as cortactin [12, 13]. These recruitment events activate the actin polymerization cellular machinery to form pedestals. Although host cells have innate mechanisms to counteract bacterial adhesion, such as the presence of inhibitory adaptor proteins [14], the bacteria ultimately adhere and damage the cells.

In children, EPEC causes persistent and chronic diarrhea lasting longer than one week [15]. Bacterial gastroenteritis is initially treated mainly through oral rehydration therapy and nutritional support, prior to administration of therapeutic agents. However, antibiotics such as second- or third-generation cephalosporins or the combination of aztreonam beta-lactams with beta-lactamase inhibitors (clavulanic acid, sulbactam, tazobactam) can be necessary for patients suffering from severe complications of gastroenteritis such as dissemination of the disease or sepsis [16].

Genetic plasticity of microorganisms and indiscriminate use of antibiotics have resulted in the development of resistant microbial strains [17]. Thus numerous studies have recently reported that diarrheagenic *E*. *coli* shows increased resistance to antibiotics [18–20]. EPEC strains isolated from the cloacae of chickens show resistance to commonly used antibacterial agents, [21], and many human EPEC isolates displays multidrug resistance to ampicillin, streptomycin, tetracyclines, trimethoprim and cotrimoxazole [22].

Natural phytocompounds can offer a new source of antibacterial agents [23, 24]. *Hibiscus sabdariffa* L. (HS) is a plant of the Malvaceae family that typically grows in subtropical and tropical areas around the world, although it seems to be native to Southeast Asia. Their calyces have been reported to be rich in dietary fiber and polyphenolic compounds [25] and are widely used to make a deep red sour tisane used in traditional medicine. Research on the physiological effects of its calyces has focused on both aqueous and organic extracts containing bioactive compounds such as tocopherols, phenolic acids, flavonoids (including anthocyanins) and organic acids (malic, oxalic, shikinic acids) [26, 27]. The potential biological activities of extracts of HS calyces are diverse and may include antibacterial properties [26, 28, 29]. *In vitro* studies have demonstrated the efficacy of HS in inhibiting pathogenic *E*. *coli* strains: aqueous, ethanolic and methanolic extracts of HS are effective against enterohemorrhagic *E*.*coli* O157:H7 (EHEC) [30, 31]. In addition, HS methanolic extract possesses bactericidal activity against clinical isolates of uropathogenic *E*. *coli* (UPEC) [32].

Acetonic, ethanolic, and methanolic extracts have shown antibacterial activity against EPEC when it was compared to chemical food sanitizers [33, 34], but these results need to be confirmed in depth and potential mechanism(s) investigated. Therefore, the present study examined the *in vitro* effects of aqueous HS extract as an antibacterial agent against EPEC, and it explored whether these effects may involve an ability to inhibit pedestal induction, since pedestals are important to EPEC pathogenicity.

## Results

### Antibacterial activity of *Hibiscus sabdariffa* extract against EPEC

**Fig 1:**
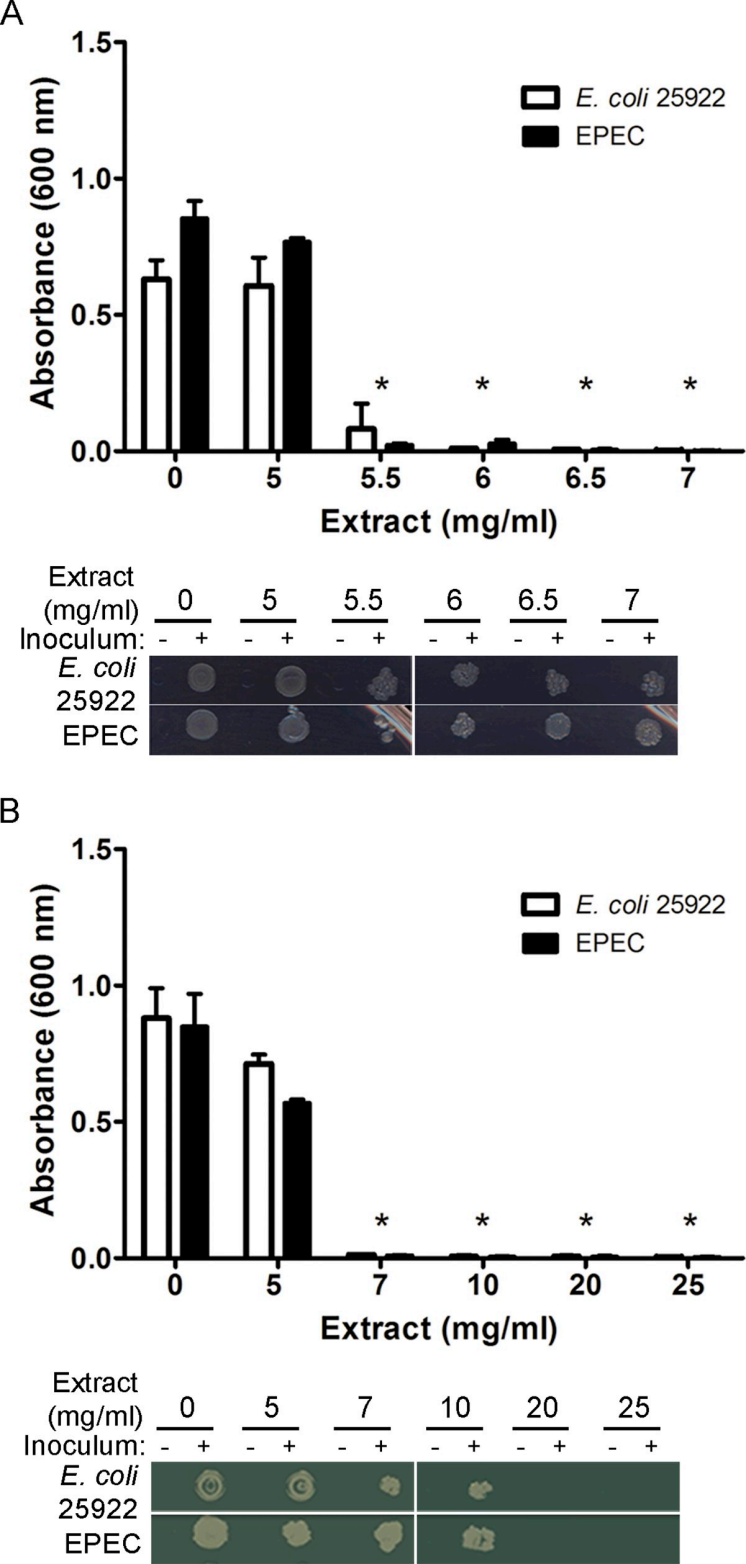
Determination of antibacterial activity of the *H*. *sabdariffa* extract against *EPEC*. **(A)** Determination of the minimum inhibitory concentration (MIC) against *E*. *coli* 25922 and EPEC. Absorbance at 600 nm of the bacterial suspension was measured after incubation at 37°C for 16–18 h with increasing concentrations of the aqueous extract. Data are mean ± SD. Statistical analysis was done using Mann Whitney test. *, p<0.05 with respect to 0 mg/ml. **(B)** Determination of the minimum bactericidal concentration (MBC) by inoculating each micro well into Mueller-Hinton Agar medium plates and incubating at 37°C for 16–18 h. The experiments were performed twice in duplicate. Data are mean ± SD. Statistical analysis was done using the Mann-Whitney test. *, p<0.05 with respect to 0 mg/ml.

The antibacterial activity of the aqueous extracts of *Hibiscus sabdariffa extract* (referred as to HS extract) was evaluated *in vitro* by broth microdilution assay to determine both the minimum inhibitory concentration (MIC) and the minimum bactericidal concentration (MBC) against enteropathogenic *E*. *coli* (EPEC). As a control, we used the recommended reference strain for antibiotic susceptibility testing, *E*. *coli* ATCC 25922 (referred as to *E*. coli). MIC values were 6.5 mg/ml against EPEC and 6 mg/ml against *E*. *coli* (Fig 1). The MBC of HS extract was estimated to be 25 mg/ml against EPEC, since growth was occasionally detected at 20 mg/ml (Fig 1).

**Fig 2:**
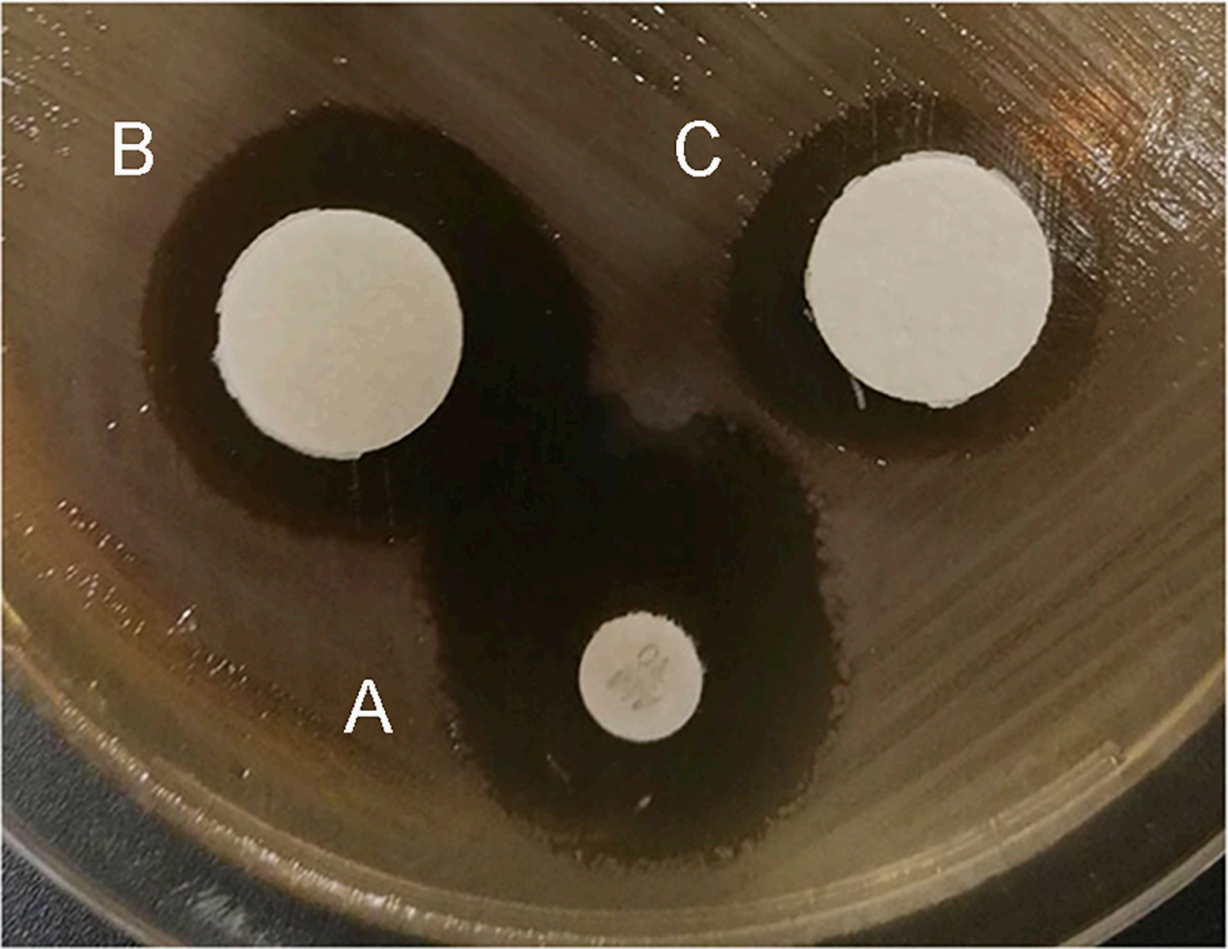
Antibacterial activity of the *H*. *sabdariffa* extract against EPEC using the disc diffusion assay. Discs contained **(A)** 10 μg ampicillin or **(B-C)** 35 mg HS extract. Representative results are shown from three experiments.

Next, the standardized Kirby-Bauer agar diffusion test for determining antimicrobial susceptibility [35] was used to confirm the antibacterial activity of HS extract against EPEC (Fig 2). Extract at a concentration of 35 mg/ml produced a halo similar in diameter to the 20-mm halo observed with a disc of 10 μg ampicillin. Extracts at concentrations of 6.5 mg/ml (MIC) did not cause detectable halos, while 25 mg/ml (MBC) produced 10 mm halos (data not shown).

#### Time-kill kinetics

**Fig 3:**
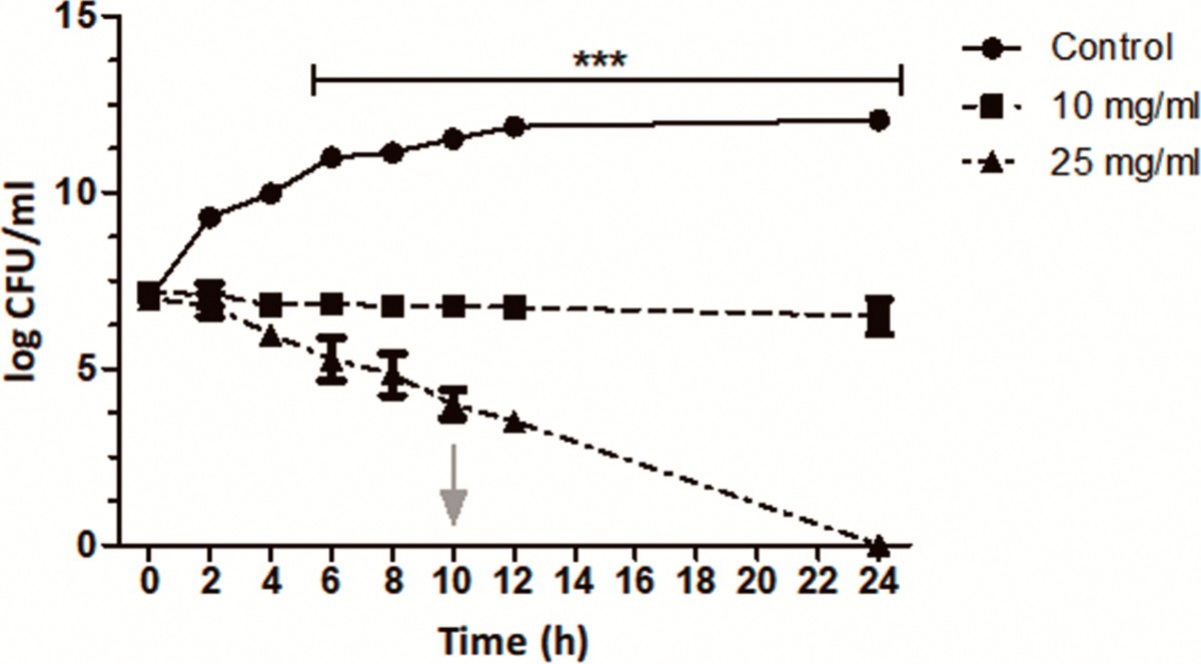
Time-kill assay of the *H*. *sabdariffa* extract against EPEC. Time-killing curves show the growth of EPEC against time in the absence (control) or presence of HS extract. The arrow indicates bactericidal activity achieved after 10-h incubation with 10 mg/ml HS extract. Results from two independent experiments were analyzed by two-way ANOVA. ***, p<0.0001.

The dynamics of bacterial killing of the HS extract were studied in time-kill kinetics assays *in vitro* at an extract concentration of 10 mg/ml, previously used in antioxidant and antimicrobial assays [27], and at the MBC of 25 mg/ml (Fig 3). We defined bactericidal activity as a decrease of at least 3 log in viable count [36]. In the time-kill assays, the extract at 25 mg/ml produced a bactericidal effect, generating a 3-log decrease in cell viability at 10 h, while no bactericidal effect was detected at 3 h. A total of 23 h was needed for the extract at 25 mg/ml concentration to achieve the bactericidal end-point (t_99.9_).

#### Effect of the treatment with the HS extract on pedestal induction by EPEC

Before analyzing the effect of HS extract on pedestal induction, we investigated possible cytotoxicity against host cells. We measured the median inhibitory dose (ID_50_), defined as the extract concentration at which 50% of cultured cells do not divide [37]. Attached HeLa cells were treated for 16 h with HS extract at 5, 7 or 10 mg/ml. The HS extract did not significantly decrease growth of HeLa cells at 5 or 7 mg/ml. However, 16 h incubation with extract at 10 mg/ml inhibited cell growth by 52% (S1 Fig). This result was confirmed with five repetitions using plates containing 1–4 x 10^5^ plated cells (data not shown).

Since the HS extract did not show antibacterial activity at 10 mg/ml (Fig 1) or significant cytotoxic activity during 3 h incubation with HeLa cells (S1 Fig), we investigated the extract's effect on pedestal induction by preactivated EPEC at a multiplicity of infection (MOI) of 10. We used extract at a concentration of 10 mg/ml rather than our experimentally determined MIC (6.5 ml/ml) because previous work used the higher concentration [27] and because the infection environment is more complex than medium containing isolated bacteria. In fact, the HS extract showed negligible bactericidal effect at 3 h (Fig 3), allowing pedestal induction.

**Fig 4:**
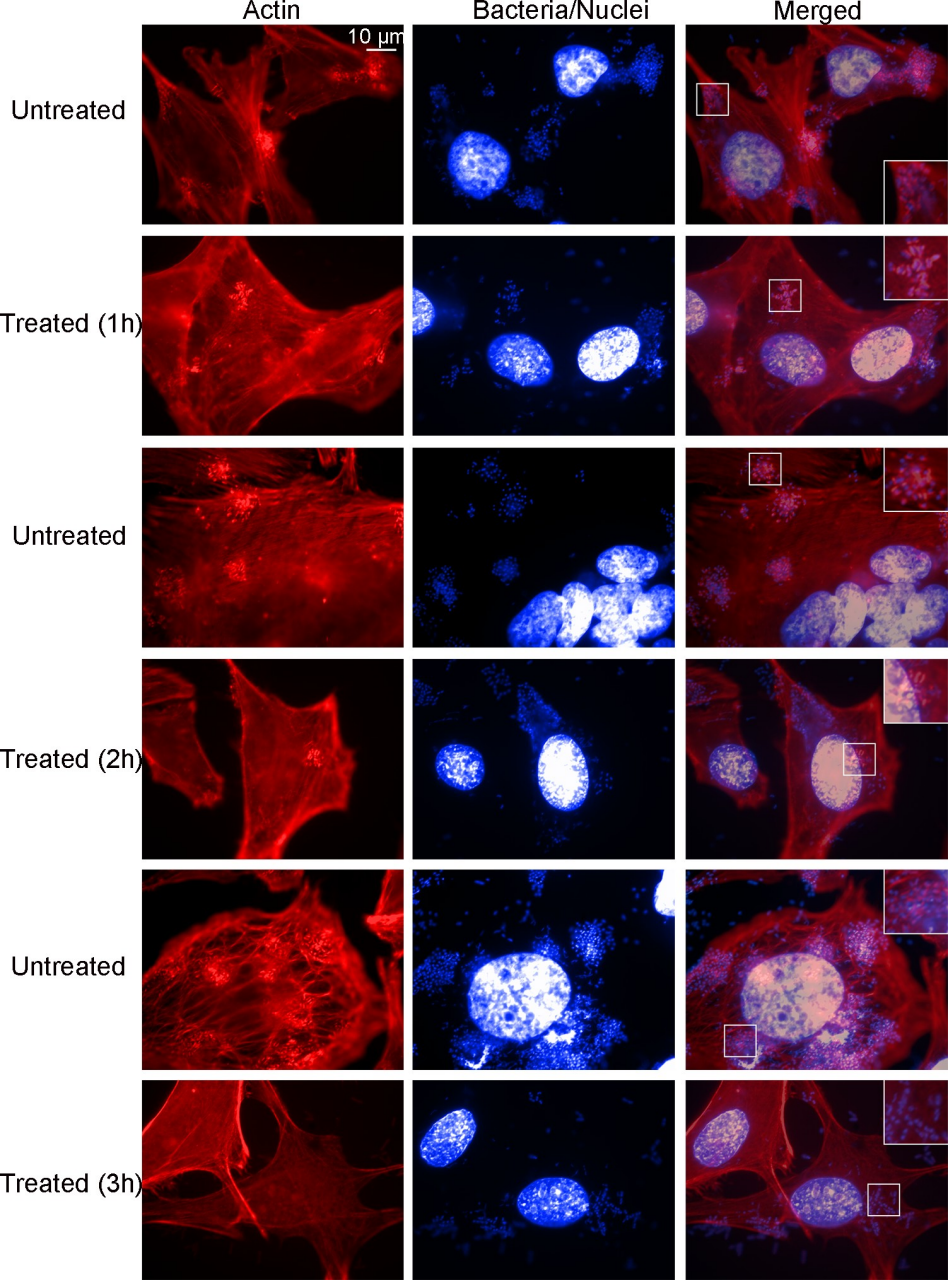
Effect of the *H*. *sabdariffa* extract on induction of pedestals by EPEC. HeLa cells were infected with EPEC at an MOI of 10 for 3 h. Unattached bacteria were washed away and the adherent cells were treated for the indicated times with the aqueous HS extract at 10 mg/ml or left untreated as a control. Then pedestals were visualized by fluorescence staining with TRITC-phalloidin (red) to stain actin and DAPI (blue) to stain bacteria. Epifluorescence micrographs were taken at a magnification of 1000 X and visualized using Adobe Photoshop. Scale bar, 10 μm. Insets, 2X digital zoom of the boxed region.

**Fig 5:**
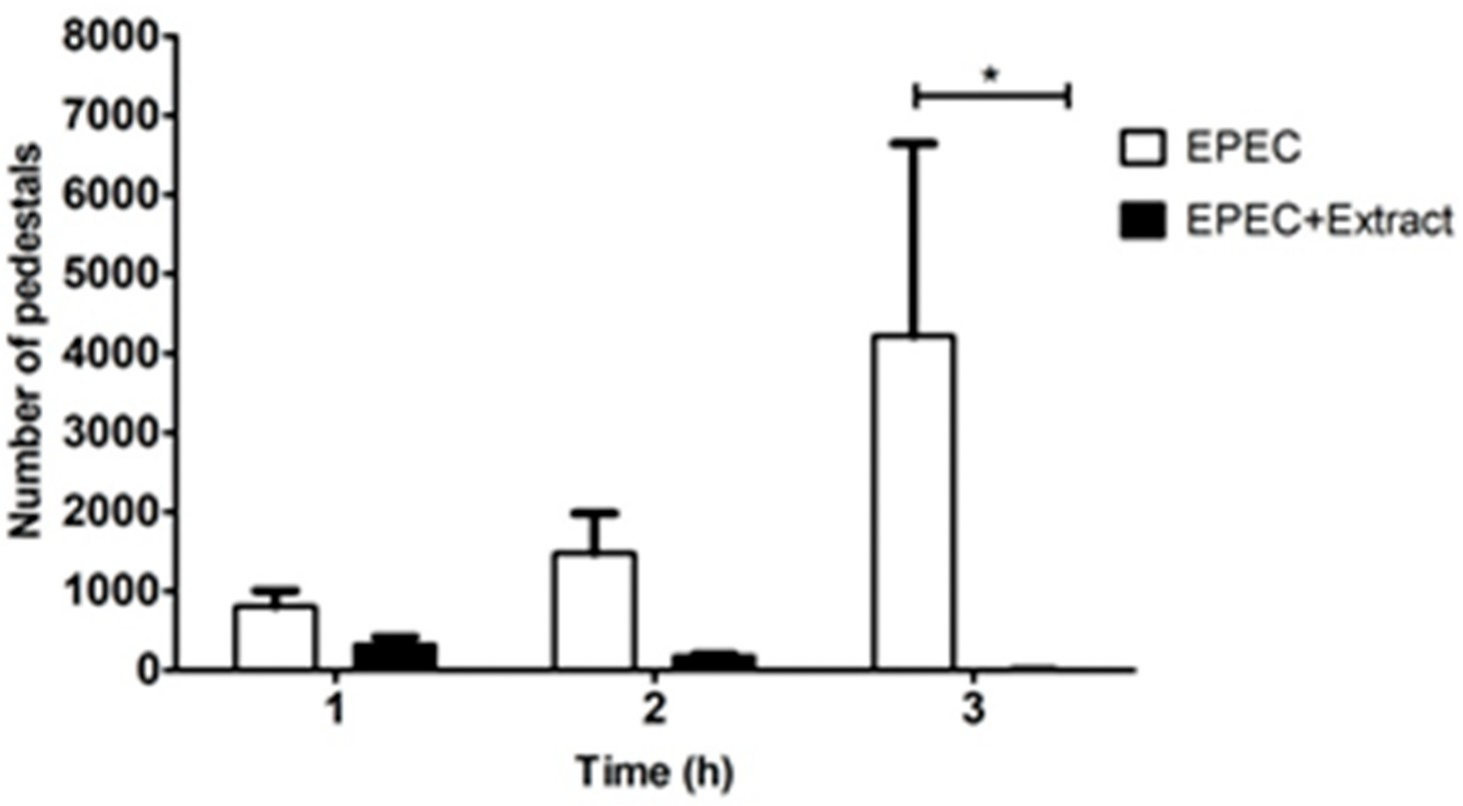
Quantitation of pedestal number. Using the assay described in Fig 4, the numbers of pedestals in HeLa cells were counted after the indicated treatments. Results from two independent experiments were analyzed using two-way ANOVA. *, p<0.014.

HeLa cells were infected for 3 h, unattached bacteria were removed, and the cells were treated with HS extract for 1, 2 and 3 h (Fig 4). Pedestal formation in treated cells, relative to the level in untreated cells, was inhibited by 59.9% at 1 h, 78.2% at 2 h, and 99.6% at 3 h (Fig 5). This result indicates that treatments with HS extract exerted significant, time-dependent inhibition of induction of pedestals by EPEC.

To determine whether the treatment with the aqueous extract prevents the induction of pedestals by EPEC, HeLa cells were pretreated for 3 hours with the HS extract and then infected with EPEC for 3 additional hours (S2 Fig). In a second set of experiments, treatments and infections were performed simultaneously (S2 Fig). In both cases, treatment with the extract reduced the formation of pedestals compared to untreated cells.

#### Effect of the treatment with the HS extract on EPEC division

**Fig 6:**
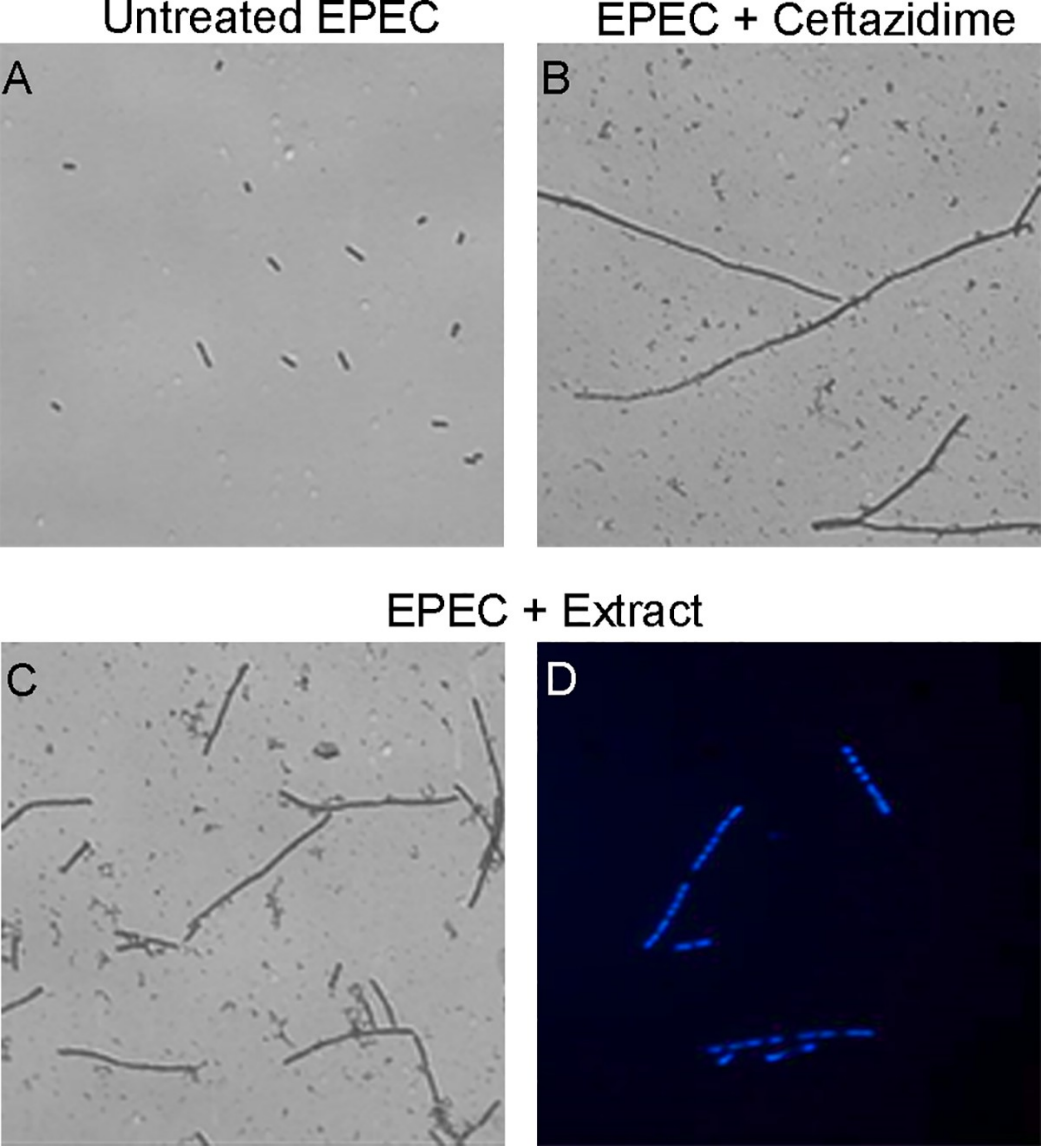
Effect of the *H*. *sabdariffa* extract on EPEC division. Crystal violet was used to stain EPEC that had been **(A)** left untreated as a control or incubated for 3 h with **(B)** ceftazidime at 0.5 μg/ml or **(C)** HS aqueous extract at 10 mg/ml. **(D)** DAPI staining was used to stain a different culture of EPEC previously incubated with HS aqueous extract at 10 mg/ml. Results are representative of three experiments.

During the EPEC infection experiments carried out in the presence of HS extract (S2 Fig), we observed under the microscope that EPEC formed filaments. It has been previously reported that the β-lactam antibiotic ceftazidime, a third-generation cephalosporin, induces a filamentous morphology of Gram-negative bacteria *E*. *coli* at sub-MIC or MIC concentrations [38]. Consequently, we compared the morphological effects of incubating EPEC with HS extract or ceftazidime for 3 h (Fig 6). We observed that the exposure of EPEC to the HS extract induced a filamentous morphology of the bacteria similar to the one induced by ceftazidime.

## Discussion

In this work, we studied the effect of *Hibiscus sabdariffa* aqueous extract on EPEC. Initially, we analyzed the potential antibacterial activity of HS extract on bacterial growth and viability by determining the MIC (6.5 mg/ml) and MBC (25 mg/ml). We indeed found that the HS extract displayed antibacterial activity against EPEC (Fig 1), which we confirmed using disc diffusion assays (Fig 2) and time-kill kinetics assays. We found that HS extract at 25 mg/ml achieved the bactericidal end-point after approximately 24 h (Fig 3).

The antimicrobial properties of ethanolic and aqueous HS extracts against Gram-positive and Gram-negative bacteria have been compared [39]. Both types of extract showed an MIC of 50 mg/ml against *E*. *coli*, based on the well diffusion method. In contrast, the aqueous extract showed a significantly lower MIC than the ethanolic extract against Gram-positive *Bacillus cereus*. Therefore based *on in vitro* studies, we conclude that the HS aqueous extract has good prospects as a antimicrobial agent against Gram-positive and Gram-negative bacteria, including important pathogens such as *Vibrio parahaemolyticus*, *Salmonella enteritidis* [39] and EPEC.

EPEC adheres to intestinal cells and induces the accumulation of polymerized actin underneath the plasma membrane of the infected cell, giving rise to a dense, actin-enriched structure called the pedestal, which has raised strong scientific interest [40]. Therefore, we next studied the effect of aqueous HS extract on pedestal induction. First we needed to assess whether the extract is cytotoxic for HeLa cells, an epithelial cell line widely used for *in vitro* EPEC infection studies. We did not detect appreciable toxic effects against HeLa cells during 3 h treatments (Fig 3), although cytotoxicity was observed after 16 h (S1 Fig). Consistent with our results, HS extracts have been reported to exert cytotoxic effects against cancer cell lines such as HeLa cells after 72 h incubation in culture medium containing 10% serum [41].

Once we established that the HS extract is not cytotoxic for HeLa cells under our infection conditions, we quantified the number of pedestals remaining after treating previously infected cells. The number of pedestals in treated cells was reduced, relative to the number in untreated cells, by 59.9% at 1 h, 78.2% at 2 h and 99.6% at 3 h, and the reduction at 3 h was statistically significant (Fig 5). To examine whether EPEC was active and possessed a functional type III secretion system, we performed Western blotting to assay the levels of Tir effector in EPEC-infected cells that were exposed or not to HS. Tir was detected in treated and untreated cells, confirming the occurrence of injection. EspF effector and actin were also detected in both types of cells. Tir, one of the most important effectors for pedestal induction by EPEC, has a rapid turnover in cells [42] and is modified by host enzymes; thus there are key post-translational modifications needed for pedestal induction including serine, threonine and tyrosine phosphorylation [43, 44]. HS treatment appears to affect the dynamics of these post-translational modifications: in untreated cells, Tir appears to be fully modified at 6 h of infection based on apparent molecular weight on SDS-PAGE gels; in treated cells, however, Tir does not appear to be fully modified although it is readily detected at 1 h (S3 Fig). This may help explain the ability of HS extract to reduce pedestal formation after 3 h of treatment (S2 Fig). Comparable findings were previously described after EPEC-infected HeLa cells were treated with proanthocyanidins from cranberry [45]. Therefore, we speculate that both treatments have an effect on host cells, which may account for the altered dynamics of Tir levels and post-translational modifications. In contrast, while our data suggest that HS extract acts directly against EPEC (Figs 1–3 and 5), such antimicrobial activity was not found for proanthocyanidins from cranberry [45].

The ability of HS to inhibit pedestal induction by EPEC implies that it may weaken bacterial adhesion to host cells, since abrogating pedestals leads to a drastic drop in bacterial binding [42]. Given the correlation between pedestal formation and bacterial adhesion [46], our findings justify further studies to examine whether HS extract may diminish bacterial attachment to cells.

Remarkably, we found that HS treatment induces EPEC filamentation, i.e. the formation of long bacterial filaments, suggesting impairment of cell division. This activity appears to be similar to that exerted by certain types of antibiotics, including ceftazidime, which we used as a positive control in our assay (Fig 5). Flavonoids may form complexes with bacterial cell walls, altering their permeability. Indeed, extracts from *Hibiscus* and other plants of known antimicrobial activity can acidify *E*. *coli* cytoplasm and hyperpolarize the bacterial cell membrane [39]. Our discovery that HS extract induces EPEC filamentation may help explain its antimicrobial effects *in vitro* and should be explored in future work.

Our findings using HS extract against EPEC are similar to results obtained against uropathogenic *E*. *coli* (UPEC) [32] and *Escherichia coli* O157:H7 (EHEC) [31]. In this way, our results and those of others establish the potential of *Hibiscus sabdariffa* extracts as an antimicrobial agent against EPEC.

## Material and methods

### Plant, bacteria, eukaryotic cells and reagents

Dried red calyces of *Hibiscus sabdariffa* var. ruber (red) purchased from a local herbalist of Hurghada city (Egypt) were originally from Sudan. The microbial quality of the calyces was checked by standard microbiology procedures [47].

For preparation of aqueous extract of HS, ground-up dried red calyces (10 g) were soaked in 100 ml of Milli Q sterile water for 24 h at room temperature. The extract was decanted and filtered using single-use, 0.22-μm sterile filters (Millipore). The filtrates were lyophilized and kept at 4°C, in the dark, until use. The yield obtained was 39%.

### Bacterial strains and growth conditions

*E*. *coli* 25922 and HeLa human epithelial cells were acquired from the American Type Culture Collection (ATCC HeLa CCL-2). Enteropathogenic *Escherichia coli* (EPEC) strain E2348/69 (0127:H6) was obtained from Dr. B. Brett Finlay (University of British Columbia, Vancouver, Canada). This strain was originally isolated in the Division of Enteric Pathogens, Central Public Health Laboratory in London from an outbreak of infantile diarrhea that occurred in Taunton (England) in 1970 [48].

Bacterial media and antibiotics were purchased from Conda-Pronadisa. Ceftazidime was used at (0.5 μg/ml). Stock cultures were maintained at 4°C on slopes of nutrient agar. Cultures for experiments were prepared by picking a single colony from 24-h Mueller-Hinton agar (MHA) plates and re-suspending it in 5 ml of Mueller-Hinton broth (MHB). Afterwards, cultures were grown aerobically for 20 h at 37°C with shaking at 200 rpm. The cultures were diluted with fresh MHB to achieve absorbance at 600 nm of 0.3, corresponding to 4 x 10^5^–2.5 x 10^8^ colony forming units (CFU) / ml based on McFarland standards.

### Determination of minimum inhibitory concentration (MIC) and minimum bactericidal concentration (MBC)

MIC and MBC were determined using the broth microwell dilution assay. The lyophilized plant extracts were dissolved (w/v) in MHB and serially diluted to concentrations ranging from 0 to 35 mg/ml or 0 to 70 mg / ml. Afterwards 95 μl of each dilution was dispensed into each well of 96-well flat-bottom microplates. Next 5 μL of the inoculum containing 2 x 10^4^ Colony Forming Units (CFU) was added to each well, except in medium growth control wells. As positive control for bacterial growth, the bacteria were grown in MHB. Plates were incubated at 37°C with shaking at 200 rpm for 16–18 h. To calculate the MIC, microbial growth was determined by reading the absorbance at 600 nm using a standard spectrophotometer. Next, to determine the MBC, 1.5 μL from each well was plated on MHA plates using a replicator and were further incubated for 24 h at 37°C (drop assay). The drop assay results were confirmed by plating the rest of the microwell. For this purpose, the microwell contents were mixed with 5 ml of MHB and incubated at 37°C with agitation for 16–18 h, then the MBC was determined by direct observation of the presence or absence of turbidity. MIC is defined as the lowest concentration of the antimicrobial compound that inhibits the growth of microorganisms after incubation. MBC is the lowest concentration of the compound that kills the bacteria.

### Kirby-Bauer agar diffusion test and time-kill kinetics assay

Antibiotic susceptibility by disc diffusion testing was realized according to the Kirby-Bauer test [35]. Sterile discs with diameters of 6 or 12 mm, depending on the amount of aqueous extract, were soaked separately in extract to obtain discs of 7, 20 or 35 mg/disc. The discs were allowed to dry, then placed on the surface of MHA plates containing EPEC at a concentration of 10^8^ CFU/ml (according to the 0.5 McFarland standard). Commercial discs of 10 μg ampicillin (BBL Taxo Discs, BD) were used as a positive control for the technique. Following 24-h incubation, the plates were inspected for an inhibition zone (halo) around the discs.

Time-kill kinetics assays for the HS extract were performed at extract concentrations of 10 and 25 mg/ml. Overnight culture bacteria were inoculated at a starting density of 4 x 10^6^ CFU/ml in MHB alone or MHB containing the HS extract. Cultures were incubated at 37°C for 24 h. Aliquots for the determination of viable counts were taken from test culture and grown for 0, 2, 4, 6, 8, 10, 12 and 24 h. Viable counts were determined by plating 100 μl of known dilutions of the culture samples onto MHA plates in duplicate. Cell count plates were incubated at 37°C for up to 48 h to allow bacterial growth. The growth curve was represented by plotting log CFU/ml against time. A 3-log unit decrease at any time point was considered to indicate bactericidal effect [36].

### HeLa cell culture

Cells were cultured in Iscove's Modified Dulbecco's Media (IMDM, Invitrogen) supplemented with 10% heat-inactivated fetal bovine serum (FBS, Lonza BioWhittaker, Thermo Fisher) and antibiotics (penicillin 100 U/ml and streptomycin 100 μg/ml, Invitrogen, Thermo Fisher) at 37°C in a humidified atmosphere with 5% CO_2_. Cell lines were routinely sub-cultured twice a week by detaching the monolayer with 0.25% trypsin—0.03% ethylene diamine tetraacetic acid (EDTA) solution. The cell passage was recorded and cells were discarded at the 25^th^ passage. Frozen cell stocks were stored in liquid nitrogen using a freezing solution containing 20% of dimethyl sulfoxide (DMSO) as a cryoprotectant.

### Cell viability assay

To determine the cytotoxicity of the extracts on HeLa cells, they were seeded at 150,000 cells per well of 6-well plates in IMDM medium supplemented with 10% heat-inactivated FBS and antibiotics and incubated at 37°C in a humidified atmosphere with 5% CO_2_ for 24 h. Then, the cells were treated with the HS extract at different concentrations (5, 7 and 10 mg/ml) for 24 h. Afterwards, the cells were washed, trypsinized and harvested in 10 ml of medium. Live cells were counted using a vital dye (trypan blue).

### Pedestal induction by EPEC

For infections, HeLa cells were grown to 70–80% confluence in 100-mm tissue culture plates in 5% CO_2_ at 37°C. Cells were then harvested, seeded at a density of 150,000 cells/well onto 6-well culture plates containing four heat-sterilized glass coverslips in each well, and allowed to attach for 24 h.

EPEC was preactivated by incubating 1:100 dilutions of an overnight Luria-Bertani broth (LB) culture for 2 h in IMDM supplemented with 10% FBS without antibiotics at 37°C and 5% CO_2_. After preactivation, the optical density of the suspension at 600 nm was adjusted to 0.2. Cells were infected at a multiplicity of infection (MOI) of 10 and allowed to form pedestals during 3 h in IMDM supplemented with 10% FBS without antibiotics.

For HS treatments, the plates were washed once with 1% FBS-IMDM, and then 1% FBS-IMDM fresh medium was added containing (or not) the HS extract at 10 mg/ml. The treatment was allowed to proceed for 1, 2 or 3 additional hours. Wells were washed with Dulbecco's PBS (D-PBS, Invitrogen) once and fixed for immunofluorescence or scraped into 200 μl standard 1X Laemmli sample buffer for Western blotting. Primary antibodies were a monoclonal antibody against Tir (2A5 MoAb) [42], obtained from Dr. B. Brett Finlay (University of British Columbia, Vancouver, Canada) [42] and a polyclonal antibody against EspF [49], obtained from Dr. Brendan Kenny (Newcastle University, UK). Monomeric actin was detected using mouse monoclonal antibody C4 against β-actin (MP Biomedicals).

### EPEC and filamentous actin staining

HeLa cells were fixed with formalin buffer solution (containing 4% w/v formaldehyde, Sigma) for 20 min at RT and then washed twice with D-PBS. The fixed cells were permeabilized with 0.05% Triton X-100 for 5 min. After washing with PBS three times, the cells were blocked with PBS containing 2% BSA for 10 min. The cells were stained with 1 μg/ml tetramethylrhodamine-isothiocyanate (TRITC)-phalloidin (Sigma) for 15 min at room temperature to visualize actin cytoskeleton and pedestals. After three washes with PBS, the cells were stained with 4'-6-diamidino-2 phenylindole (DAPI, 300 nM) for 5 min at room temperature to visualize bacteria. After three washes with PBS, the coverslips were allowed to dry at room temperature during 20 min and mounted using 5 μl of mounting medium containing 100 mM Tris-HCl (pH 8.5), 10% Mowiol 4–88 (Calbiochem), and 25% glycerol with phenylnediamine (Sigma). Micrographs were taken using a Nikon Eclipse TE 200-U fluorescence microscope equipped with a Hamamatsu camera. Images were processed with Adobe Photoshop. Quantification of pedestals was done by counting the number of pedestals of attached bacteria for a total of 40 infected cells in representative microscope fields.

## Supporting information

S1 Fig Effect of the *H*. *sabdariffa* extract on HeLa cell growth. The number of viable HeLa cells was counted after 16 h incubation in the absence (white) or presence (colors) of HS extract at the indicated concentrations. Results from two independent experiments are shown and analyzed using Student´s *t* test. *, p<0.05.
(TIF)

S2 Fig Induction of pedestals by EPEC depending on the timing of treatment with *H*. *sabdariffa* extract. **(A)** HeLa cells were treated for 3 h with the HS extract or left untreated as a negative control. Then the cells were washed and subsequently infected with EPEC for 3 h. **(B)** HS extract and EPEC were simultaneously added to HeLa cells and infections were allowed to proceed for 3 h. As a control, HeLa cells were infected with EPEC for 3 h. EPEC was added at an MOI of 10. Pedestals were visualized by fluorescence staining with TRITC-phalloidin (red) to stain actin and DAPI to stain bacteria (blue). Epifluorescence micrographs were taken at a magnification of 1000X and visualized using Adobe Photoshop. Representative results are shown from three experiments. Scale bar, 10 μm. Insets, 2X digital zoom of the boxed regions.
(TIF)

S3 Fig Tir and EspF levels in HeLa cells after treatment with the *H*. *sabdariffa* extract. HeLa cells were infected for 3 h and then left untreated or treated with HS extract for 1, 2 and 3 additional hours. Cell lysates were analyzed by Western blotting using a monoclonal antibody (MoAb) against Tir or a polyclonal antibody (Ab) against EspF. Actin was detected as a loading control.
(TIF)
