# Supplementary figures and images for: Aqueous extract of Hibiscus sabdariffa inhibits pedestal induction by enteropathogenic E. coli and promotes bacterial filamentation in vitro
Source: PLoS One. 2019 Mar 8;14(3):e0213580. doi: 10.1371/journal.pone.0213580 (PMC6407759; doi:10.1371/journal.pone.0213580)

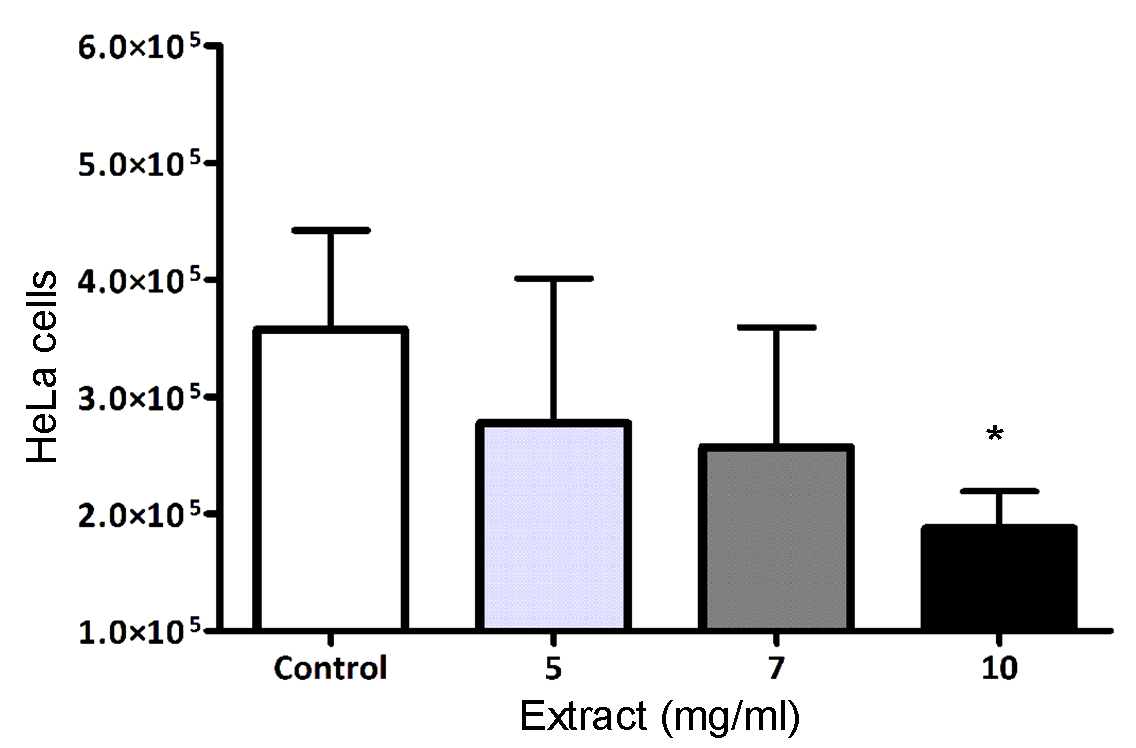

Supplement: S1 Fig — The number of viable HeLa cells was counted after 16 h incubation in the absence (white) or presence (colors) of HS extract at the indicated concentrations. Results from two independent experiments are shown and analyzed using Student´s t test. *, p<0.05. (TIF) [file pone.0213580.s001.tif]

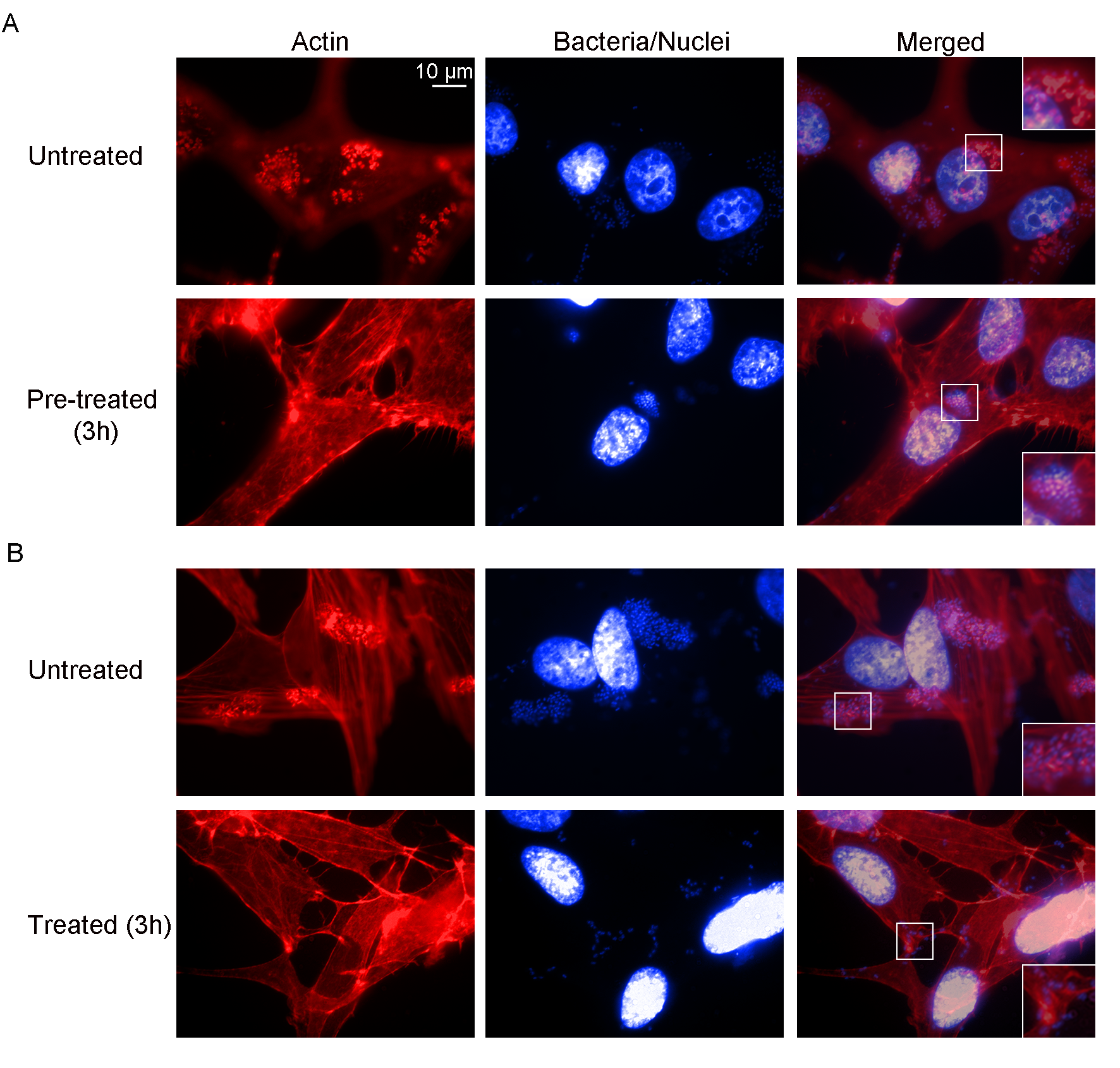

Supplement: S2 Fig — (A) HeLa cells were treated for 3 h with the HS extract or left untreated as a negative control. Then the cells were washed and subsequently infected with EPEC for 3 h. (B) HS extract and EPEC were simultaneously added to HeLa cells and infections were allowed to proceed for 3 h. As a control, HeLa cells were infected with EPEC for 3 h. EPEC was added at an MOI of 10. Pedestals were visualized by fluorescence staining with TRITC-phalloidin (red) to stain actin and DAPI to stain bacteria (blue). Epifluorescence micrographs were taken at a magnification of 1000X and visualized using Adobe Photoshop. Representative results are shown from three experiments. Scale bar, 10 μm. Insets, 2X digital zoom of the boxed regions. (TIF) [file pone.0213580.s002.tif]

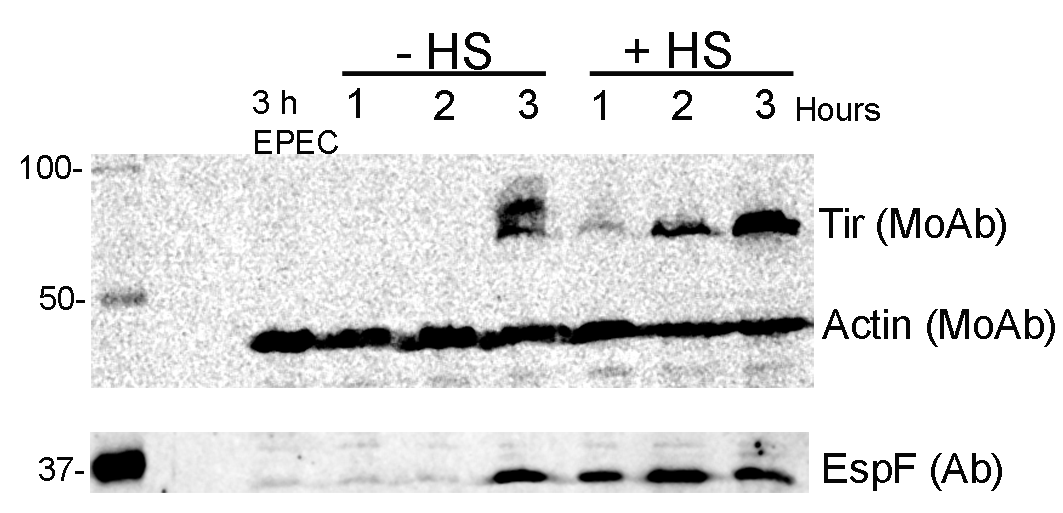

Supplement: S3 Fig — HeLa cells were infected for 3 h and then left untreated or treated with HS extract for 1, 2 and 3 additional hours. Cell lysates were analyzed by Western blotting using a monoclonal antibody (MoAb) against Tir or a polyclonal antibody (Ab) against EspF. Actin was detected as a loading control. (TIF) [file pone.0213580.s003.tif]
